# *Candida* species Rewired Hyphae Developmental Programs for Chlamydospore Formation

**DOI:** 10.3389/fmicb.2016.01697

**Published:** 2016-10-27

**Authors:** Bettina Böttcher, Christine Pöllath, Peter Staib, Bernhard Hube, Sascha Brunke

**Affiliations:** ^1^Department of Microbial Pathogenicity Mechanisms, Hans-Knöll-InstituteJena, Germany; ^2^Institute for Medical Microbiology, Jena University HospitalJena, Germany; ^3^Center for Sepsis Control and Care, Jena University HospitalJena, Germany; ^4^Department of Research and Development, Kneipp GmbHWürzburg, Germany; ^5^Friedrich Schiller University JenaJena, Germany

**Keywords:** *Candida*, morphology, chlamydospores, Tor pathway, starvation, fungal pathogens

## Abstract

Chlamydospore formation is a characteristic of many fungal species, among them the closely related human-pathogenic dimorphic yeasts *Candida albicans* and *C. dubliniensis*. Whereas function and regulation of filamentation are well-studied in these species, the basis of chlamydospore formation is mostly unknown. Here, we investigate the contribution of environmental and genetic factors and identified central proteins involved in species-specific regulation of chlamydosporulation. We show that specific nutrient levels strongly impact chlamydospore initiation, with starvation favoring sporulation and elevated levels of saccharides or peptone inhibiting it. Thresholds for these nutritional effects differ between *C. albicans* and *C. dubliniensis*, which explain species-specific chlamydospore formation on certain diagnostic media. A *C. albicans nrg1*Δ mutant phenocopied *C. dubliniensis*, putting Nrg1 regulation at the basis of species-specific chlamydospore formation under various conditions. By screening a series of potential chlamydospore regulators, we identified the TOR and cAMP pathways as crucial for sporulation. As rapamycin treatment blocked chlamydosporulation, a low basal Tor1 activity seems to be essential. In addition, TOR effector pathways play an important role, and loss of the NCR (nitrogen catabolite repression) gene regulators Gat1 and Gln3 reduced chlamydospore formation. A severe reduction was seen for a *C. albicans gcn4*Δ deletion strain, implicating a link between regulation of amino acid biosynthesis and chlamydospore development. On the other hand, deletion of the GTPase gene *RAS1* and the adenylyl cyclase gene *CYR1* caused a defect in chlamydospore formation that was mostly rescued by cAMP supplementation. Thus, cAMP-signaling is a second major pathway to control chlamydospore production. Finally, we confirmed light exposure to have a repressive effect on chlamydosporulation. However, permanent illumination only reduced, but not abolished chlamydospore production of *C. albicans* whereas *C. dubliniensis* sporulation was unaffected. In summary, we describe novel environmental factors which determine chlamydosporulation and propose a first model for the regulatory network of chlamydospore formation by different *Candida* species.

## Introduction

Morphological transitions are a defining feature of polymorphic yeasts, including some *Candida* species which are important pathogens of humans. These changes in cell shape can usually be induced by a number of different stimuli, including temperature, pH value changes, oxygen level, and availability of nutrients. Current research on *Candida* morphology focuses mainly on the yeast-to-hypha-transition, a key virulence factor especially of *C. albicans* (Mayer et al., 2013). In comparison, another morphological structure, the chlamydospores—thick-walled, globular structures formed via suspensor cells on hyphal tips—is often neglected and their biological function remain a mystery. However, they are used as a reliable and cheap diagnostic tool (Campanha et al., 2005), as in addition to certain *Candida* species, many different fungi like *Fusarium*, the dermatophytes, or *Cryptococcus* can form chlamydospores. Their appearence on specific media can hence often serve as a first criterion for fungal species identification (Bakerspigel, 1954; Park, 1954; Lin and Heitman, 2005). Within the *Candida* clade, the close relatives *C. albicans* and *C. dubliniensis* are the only known producers of chlamydospores in addition to other morphological forms, such as yeasts, pseudohyphae, and true hyphae (Staib and Morschhäuser, 2007; Moran et al., 2012).

In general, vegetative spores are formed as resting structures by many soil-borne fungi under nutrient-limited conditions. However, in *Candida* the term chlamydospore does not refer to a functional, but rather to a morphological unit: Although *Candida* chlamydospores resemble such spores and are high in lipid droplets for energy supply (Jansons and Nickerson, 1970a), they are not more resistant to heat, starvation, or dryness compared to yeast cells (Citiulo et al., 2009). Nevertheless, early studies reported a correlation between nutrient supply and induction of chlamydospore formation of the fungus (Jansons and Nickerson, 1970b) where glucose, in contrast to nitrogen, had a strong repressive effect (Dujardin et al., 1980b). Typical inducing media on the other hand are rich in complex carbon sources (e.g., corn or rice meal) and frequently contain detergents. Importantly, *C. dubliniensis* has a higher propensity to produce chlamydospores under conditions where *C. albicans* largely remains in yeast form, and many diagnostic media use this characteristic to differentiate between the two species (Staib and Morschhäuser, 1999). Furthermore, under such conditions where *C. albicans* and *C. dubliniensis* form chlamydospores, *C. tropicalis* robustly grows as pseudohyphae without any chlamydospores, *C. parapsilosis* grows as short filaments, and *C. glabrata* as yeasts (Silva et al., 2012). This hints toward a conserved morphogenetic pathway between *C. albicans* and *C. dubliniensis*, which is not present in more distantly related non-*albicans Candida* species.

The natural niche for chlamydospore formation remains unknown, and no clear role of chlamydospores for the commensal or pathogenic life style of *Candida* has been demonstrated: Only sporadic studies reported the isolation of chlamydospores from candidemia patients (Chabasse et al., 1988) and some *C. albicans* isolates from clinical specimens were found to be chlamydospore-negative (Al-Hedaithy and Fotedar, 2002). Thus, a possible involvement of chlamydospores in the infection process is unclear. However, the fact that the ability to form chlamydospores has been conserved in the vast majority of *C. albicans* and *C. dubliniensis* isolates suggests an important biological function for both species. Although the biological function of chlamydospores remains to be uncovered, essential genes required for their formation have been described (Nobile et al., 2003). Some of these genes are part of the stress-responsive HOG and Cek1-mediated MAPK pathways (Sonneborn et al., 1999; Eisman et al., 2006), while others form a loose network without clear connections to other signal transduction pathways in *Candida* spp. (Nobile et al., 2003).

Fundamental regulatory differences are known for the other morphologies of *C. albicans* and *C. dubliniensis*. In contrast to *C. albicans, C. dubliniensis* rarely forms true hyphae, which has been linked to modified hyphae-promoting regulatory pathways, namely the Tor1 and cAMP-PKA cascades (Sullivan and Moran, 2011; Caplice and Moran, 2015). Species-specific thresholds for nutritional signals seem to define the unique gene expression patterns, which are finally controlled by the central transcription factors Nrg1, Efg1, and Ume6 (Moran et al., 2007; O'Connor et al., 2010). Despite few recent studies on the regulation of chlamydospore formation, most research papers dealing with *Candida* chlamydospores were published before the discovery of *C. dubliniensis* in 1995 (Sullivan et al., 1995) and detailed information on *Candida* species-specific factors of chlamydosporulation is largely lacking. Nevertheless, the major hyphae repressor Nrg1 seems to play a key role in the suppression of chlamydosporulation of *C. albicans*. Therefore, Nrg1 is considered one of the most important factors for the species-specific characteristics of yeast-to-hypha transitions (Moran et al., 2007) as well as chlamydospore formation (Staib and Morschhäuser, 2005).

In this study, we re-evaluated the idea of a nutritional control of chlamydospore development, systematically compared *C. albicans*- and *C. dubliniensis*-specific features of chlamydospore induction under a range of conditions, and screened a series of mutants lacking key regulators of morphology in combination with specific inhibitors of regulatory pathways. Based on these data, we propose a model of chlamydospore regulation that combines rewiring of hyphae-specific pathways and chlamydospore- as well as species-specific aspects.

## Materials and methods

### Strains and culture conditions

*Candida* strains and mutants are listed in Table 1 and were routinely propagated on YPD agar (10 g yeast extract, 20 g peptone, 20 g glucose, 15 g agar per liter) at 30°C and stored as frozen stocks in YPD medium with 15% (v/v) glycerol at −80°C.

**Table 1:** *****Candida*** strains used in this work**.

**Strain**	**Parent**	**Genotype**	**References**
***C. albicans*** **STRAINS**			
SC5314		*C. albicans* wild-type strain	Gillum et al., 1984
RM1000	CAI4	*ura3*Δ*::*λ*imm434/ura3*Δ*::*λ*imm434 his1*Δ*::hisG/his1*Δ*::hisG rps1::URA3 IRO1/iro1*Δ*::*λ*imm434*	Negredo et al., 1997
CAI4	SC5314	*ura3*Δ*::*λ*imm434/ura3*Δ*::*λ*imm434 IRO1/iro1*Δ*::*λ*imm434*	Fonzi and Irwin, 1993
BWP17	RM1000	*ura3*Δ*::*λ*imm434/ura3*Δ*::*λ*imm434 IRO1/iro1*Δ*::*λ*imm434 his1*Δ*::hisG/his1*Δ*::hisG arg4*Δ*/arg4*Δ	Wilson et al., 1999
DAY286	RM1000	*ura3*Δ*::*λ*imm434/ura3*Δ*::*λ*imm434 pARG4::URA3::arg4*Δ*::hisG/arg4*Δ*::hisG his1*Δ*::hisG/his1*Δ*::hisG*	Davis et al., 2002
THE1	CAI4	*ade2*Δ*::hisG/ade2*Δ*::hisG ura3*Δ*::*λ*imm434/ura3*Δ*::*λ*imm434 ENO1/eno1*Δ*::ENO1-tetR-ScHAP4AD-3 × HA-ADE2 ade2*Δ*::hisG/ade2*Δ*::ADE2-URA3-97t-RLUC1*	Nakayama et al., 2000
*nrg1*Δ	CAI4	*ura3*Δ*::*λ*imm434/ura3*Δ*::*λ*imm434 IRO1/iro1*Δ*::*λ*imm434 nrg1*Δ*::hisG-URA3-hisG/nrg1*Δ*::hisG*	Murad et al., 2001
*suv3*Δ	BWP17	*ura3*Δ*::*λ*imm434/ura3*Δ*::*λ*imm434 IRO1/iro1*Δ*::*λ*imm434 arg4*Δ*::hisG/arg4*Δ*::hisG his1*Δ*::hisG/his1*Δ*::hisG suv3*Δ*::Tn7-UAU1/suv3*Δ*::Tn7-URA3*	Nobile and Mitchell, 2009
*sch9*Δ	BWP17	*ura3*Δ*::*λ*imm434/ura3*Δ*::*λ*imm434 IRO1/iro1*Δ*::*λ*imm434 arg4*Δ*::hisG/arg4*Δ*::hisG his1*Δ*::hisG/his1*Δ*::hisG sch9*Δ*::Tn7-UAU1/ sch9*Δ*::Tn7-URA3*	Nobile and Mitchell, 2009
*mds3*Δ	BWP17	*ura3*Δ*::*λ*imm434/ura3*Δ*::*λ*imm434 IRO1/iro1*Δ*::*λ*imm434 arg4*Δ*::hisG/arg4*Δ*::hisG his1*Δ*::hisG/his1*Δ*::hisG mds3*Δ*::Tn7-UAU1/ mds3*Δ*::Tn7-URA3*	Nobile and Mitchell, 2009
*rim101*Δ	CAI4	*rim101*Δ*::hisG/rim101*Δ*::hisG-URA3-hisG IRO1/iro1*Δ*::*λ*imm434*	Ramon et al., 1999
*vps11*Δ	BWP17	*his1*Δ*/his1*Δ*::HIS1 vps11*Δ*::ARG4/vps11*Δ*::URA3 IRO1/iro1*Δ*::*λ*imm434*	Palmer et al., 2003
*dit2*Δ	CAI4	*dit2*Δ*::HIS1/dit2*Δ*::URA3 his1*Δ*/his1*Δ*ura3*Δ*::*λ*imm434/ura3*Δ*::*λ*imm434 IRO1/iro1*Δ*::*λ*imm434*	Arnold Bito, University of Salzburg, Austria
*sit4*Δ^*^	RM1000	*sit4*Δ*::HIS1/sit4*Δ*::dpl200 rps1::URA3 IRO1/iro1*Δ*::*λ*imm434*	Lee et al., 2004
*gcn2*Δ^*^	CAI4	*gcn2*Δ*::hisG/gcn2*Δ*::hisG rps1::URA3 IRO1/iro1*Δ*::*λ*imm434*	Tournu et al., 2005
*gcn4*Δ	CAI4	*ura3*Δ*::*λ*imm434/ura3*Δ*::*λ*imm434, gcn4*Δ*::hisG-URA3-hisG/gcn4::hisG IRO1/iro1*Δ*::*λ*imm434*	Tripathi et al., 2002
*gat1*Δ	CAI4	*gat1*Δ*::hisG/gat1*Δ*::hisG iro1-ura3*Δ*::*λ*imm434/IRO1-URA3*	Limjindaporn et al., 2003
*gln3*Δ	CAI4	*gln3*Δ*::hisG/gln3*Δ*::hisG iro1-ura3*Δ*::*λ*imm434/IRO1-URA3*	Liao et al., 2008
*cyr1*Δ	CAI4	*cyr1*Δ*::hisG/cyr1*Δ*::hisG iro1-ura3*Δ*::*Δ*imm434/iro1-ura3*Δ*::*λ*imm434*	Rocha et al., 2001
*pde2*Δ	SC5314	*pde2*Δ*::FRT/ pde2*Δ*::FRT*	Yi et al., 2011
*ras1*Δ	CAI4	*ras1*Δ*::hisG/ras1*Δ*::hph-URA3-hph IRO1/iro1*Δ*::*λ*imm434*	Feng et al., 1999
*ume6*Δ	SN87	*ume6*Δ*::CdHIS1/ume6*Δ*::CmLEU2 IRO1/iro1*Δ*::*λ*imm434*	Zeidler et al., 2009
*UME6*^OE^**	THE1	*ume6*Δ*::P_TET*_OFF_*-UME6/ume6*Δ*::FRT*	Melanie Polke, Hans-Knöll-Institut, Jena, Germany
***C. dubliniensis*** **STRAIN**			
Wü284		*C. dubliniensis* wild-type strain	Morschhäuser et al., 1999

* *To restore prototrophy, strains based on CAI4 were complemented with URA3 using CIp10*.

### Strain constructions

All strains are listed in Table 1. The uridine auxotrophic CAI4-based *C. albicans* strains were complemented with *URA3* by integration of a *Stu*I-linearized CIp10 vector (Murad et al., 2000). Transformations were performed following standard procedures (Walther and Wendland, 2003). Briefly, cultures were washed and treated with lithium-acetate solution, the linearized vector was added together with carrier DNA and polyethylene glycol, and the cells were exposed to a 15 min heat shock at 44°C. Uridine-positive transformants were selected on SD agar. Integration at the *RPS1* locus was confirmed by PCR.

### Growth curve analyses

The effect of rapamycin on cell proliferation was evaluated via growth curve assays. Strains were pre-grown overnight in YPD at 30°C and after repeated washing they were diluted to OD_600_ = 0.01 in YPD with and without supplementation of 5 nM rapamycin (Sigma-Aldrich and Merck KGaA, Darmstadt, Germany) solved in DMSO (Carl Roth GmbH & Co. KG, Karlsruhe, Germany) or an adequate amount of DMSO as control. Cultures were incubated at 30°C in a Magellan TECAN plate reader (Tecan I-Control Infinite® 200 Pro, Tecan Austria GmbH, Grödig, Austria) with the extinction in the wells at 600 nm determined over 48 h every 15 to 30 min after 30 s orbital shaking. Generation times as the time for one doubling of cell count was calculated in phases of exponential growth (Hall et al., 2014). Additionally, the initial time points and the extinction in the wells at 600 nm at the stationary phase were measured.

### Chlamydospore formation

Chlamydospore production was induced on corn meal (CM)-Tween 80 agar (BD, Heidelberg, Germany), rice meal-Tween 80 agar (BD, Heidelberg, Germany), yeast nitrogen base medium (YNB) without ammonium sulfate (MP, Santa Ana, CA, USA) agar, or SLAD agar (YNB without ammonium sulfate, with 2% glucose), each supplemented as indicated. The plates were incubated at 27°C for 7 days in darkness and chlamydospore formation was monitored microscopically (Axiovert, Zeiss, Göttingen, Germany). For quantification of chlamydospores a score system (chlamydospore index, CI) was introduced with a scale ranging from none (0), intermediate (1–2) to full (3) chlamydospore production (see Supplementary Figure S1 for details).

### Inhibition of chlamydospore formation

Different substances were tested for their influence on chlamydospore formation. Additional carbon sources (glucose, maltose, lactose, sucrose, galactose, glycerol, and mannitol), ammonium sulfate, peptone, caffeine (Sigma-Aldrich and Merck KGaA, Darmstadt, Germany) or heat-inactivated fetal calf serum (Bio&SELL GmbH, Feucht, Germany) were directly added to the liquid agar at 60°C. Dibutyryl-cAMP (db-cAMP; Sigma-Aldrich and Merck KGaA, Darmstadt, Germany), rapamycin (solved in DMSO), or DMSO (vehicle control) were distributed on top of solid agar plates to avoid heat-induced degradation. Effects of inhibitors were tested with agar diffusion assays. To this end, an 8 mm diameter hole in the agar plate was filled with liquid test substances. After the liquid was completely soaked into the agar, the *Candida* strains were streaked radially from the agar hole.

### Protein alignment

Protein light sensors (IreA, IreB and VeA) of *Aspergillus nidulans* were aligned to the proteome of *C. albicans* SC5314 and *C. dubliniensis* Wü284 using the PBLAST tool at the *Candida* Genome Database (http://www.candidagenome.org).

## Results and discussion

### Nutrient availability effects on chlamydospore formation depend on the species

**Figure 1:**
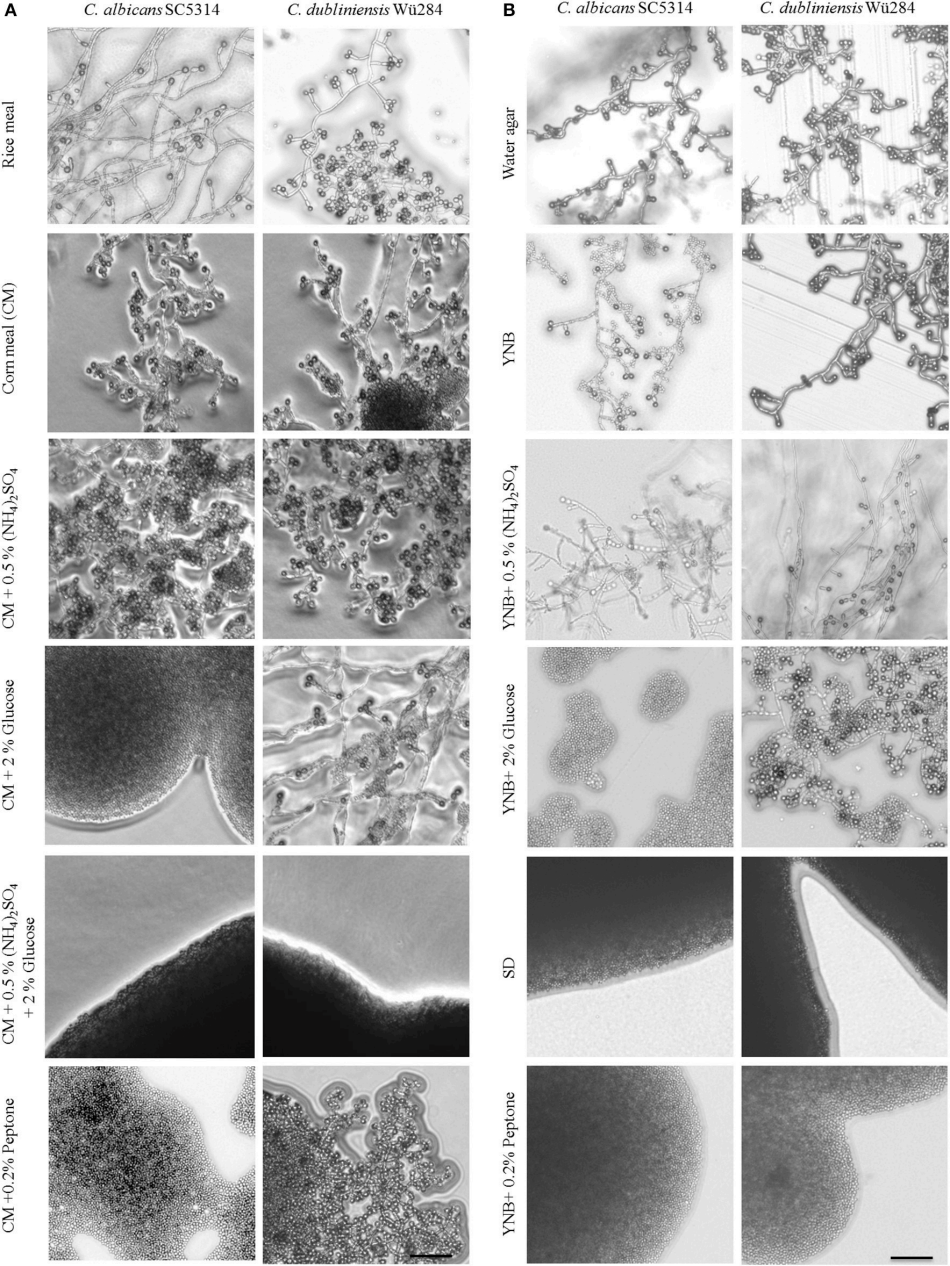
**Nutrients influence chlamydospore formation of ***C. albicans*** and ***C. dubliniensis*****. *Candida* wild type strains were streaked on **(A)** rice or corn meal (CM) or **(B)** YNB agar supplemented with ammonium sulfate, glucose, or peptone. On day 7 of incubation (27°C, darkness), colony morphology was examined microscopically. Both species produced typically high amounts of chlamydospores on rice and CM agar (chlamydospore index [CI] 3). On water and YNB agar in absence and presence of ammonium sulfate sporulation by *C. dubliniensis* was strong (CI 3) and at an intermediate level for *C. albicans* (CI 1–2). Addition of peptone or a combination of glucose and ammonium sulfate inhibited sporulation of both species. 2% glucose as a sole supplement blocked chlamydospore formation of *C. albicans*, but not *C. dubliniensis*. Scale bar, 100 μm.

Nutrient availability controls morphogenesis in a diverse range of fungi. For example, next to mating type, lack of nitrogen or fermentable carbon sources is the most important factor to induce sexual sporulation of *Saccharomyces cerevisiae* (Mitchell, 1994). Fungal secondary metabolism (Tudzynski, 2014) as well as many pathogenicity pathways (Limjindaporn et al., 2003) are similarly responsive to nitrogen availability. Morphological transitions that are triggered by nitrogen starvation include pseudohyphal growth of diploid *S. cerevisiae* (Gimeno et al., 1992) and the Gcn4-dependent filamentation by *C. albicans* (Tripathi et al., 2002). Some impact of nitrogen on chlamydosporulation has also been described for *C. albicans* (Jansons and Nickerson, 1970b; Dujardin et al., 1980b) but no such information is available for *C. dubliniensis* so far. Hence, we tested the effect of nutrient addition to chlamydospore-inducing corn meal tween (CM) agar and rice agar. The results are shown in Table 2 using the chlamydospore index (CI) as a semi-quantitative score system (see Figure S1 for representative micrographs of different scores). Presence of additional 0.5% ammonium sulfate had no effect on the strong chlamydospore formation of both, *C. albicans* and *C. dubliniensis*, on these media (Figure 1A). Addition of a readily available carbon source (2% glucose) specifically inhibited *C. albicans* chlamydosporulation. However, only the presence of both, additional carbon and nitrogen sources, reduced chlamydospore formation in *C. dubliniensis*.

**Table 2:** **Effects of different carbon and nitrogen sources on chlamydospore formation by ***C. albicans*** SC5314, ***C. dubliniensis*** Wü284, and ***C. albicans nrg1***Δ**.

**Agar**	***C. albicans* SC5314**	***C. dubliniensis* Wü284**	***C. albicans nrg1Δ***
Cornmeal (CM)	3	3	3
+ 2% Glucose	0	1	3
+ 0.5% Ammonium sulfate	3	3	3
+ 2% Glucose, 0.5% ammonium sulfate	0	0	0
+ 2% Galactose	0	1.5	2
+ 2% Lactose	3	3	3
+ 2% Sucrose	0	3	3
+ 2% Maltose	0	1	3
+ 2% Mannitol	1	3	3
+ 2% Glycerol	3	3	3
+ 0.2% Peptone	0	2	3
+ 2% Peptone	0	0	1.5
Water agar	2	3	3
Yeast nitrogen base (YNB)	1.5	3	3
+ 2% Glucose (SLAD)	0	3	3
+ 0.5% Ammonium sulfate	1	3	3
+ 2% Glucose, 0.5% ammonium sulfate (SD)	0	0	0
+ 2% Galactose	0	2.5	3
+ 2% Lactose	1	3	3
+ 2% Sucrose	0	3	3
+ 2% Mannitol	0	2.5	3
+ 2% Glycerol	1.5	2	3
+ 0.2% Peptone	0	0	3
+ 2% Peptone	0	0	0

*Numbers indicate chlamydospore quantity (CI), with 3 = strong, 1–2 = little to medium, and 0 = no sporulation. See Figure S1 for exemplary micrographs of colony morphologies*.

To elucidate this effect further, we used defined synthetic media. Pure water agar as a highly nutrient-restricted medium induced chlamydospores in both, *C. albicans* and *C. dubliniensis* [(Jansons and Nickerson, 1970b) and Figure 1B]. This was not visible on standard, N- and C-source containing SD agar, suggesting that nutrient supplementation inhibits sporulation. In order to distinguish between nitrogen-, carbon-, and other nutrient-responsive effects, a minimal medium—yeast nitrogen base (YNB)—was used to provide only essential trace metals, vitamins, and minerals. This medium readily induced chlamydospores in both species (Figure 1B), and we thus excluded a major sporulation effect by the presence of micronutrients alone. The addition of 2% glucose to YNB (a medium equal to SLAD agar) blocked chlamydospore formation again specifically in *C. albicans*. Interestingly, SLAD agar is a medium well-known to induce hyphae-like and invasive growth in both, *C. albicans* and *S. cerevisiae* (Cutler et al., 2001; Laxman and Tu, 2011), which may indicate an evolutionary connection between pseudohypha and chlamydospore formation pathways. YNB agar supplementation with 0.5% ammonium sulfate dramatically reduced, but did not abolish sporulation by *C. albicans*. Similar to CM agar, only a combination of nitrogen and carbon sources inhibited sporulation of *C. dubliniensis*, while addition of a single macronutrient had little or no inhibiting effect.

In contrast to ammonium, the addition of even low concentrations of peptone as an organic nitrogen source (0.2%) had a strong repressive effect on chlamydospore formation, independent of the *Candida* species (Figure 1B). This observation indicates a crucial role of amino acid presence and catabolism rather than just total nitrogen content on sporulation. Previous transcriptome analysis under chlamydospore-inducing conditions showed a significant up-regulation of peptide starvation response genes, e.g., the oligopeptide transporter gene *PTR2* (Palige et al., 2013). Ptr2 is a conserved high-affinity transporter which was shown to physically interact with the nutrient-sensing Tor1 and Tor2 complexes in *S. cerevisiae* (Aronova et al., 2007). This Ptr2 up-regulation and the inhibitory effect of peptone thus indicate that sensing of peptide starvation is a major chlamydospore-inducing factor. However, in CM agar, peptone inhibited chlamydospores only in *C. albicans*. Therefore, in comparison to YNB agar, CM agar seems to be a generally stronger trigger of chlamydospore formation, able to induce robust sporulation by both *Candida* species and showing less inhibition by the presence of nutrients for *C. dubliniensis*.

Another species-specific repressing role of peptone, here on filamentation, has been described earlier: In the presence of peptone, *UME6* gene expression is low in *C. dubliniensis*, but high in *C. albicans*, allowing hyphae formation only by the latter (O'Connor et al., 2010). Artificial overexpression of *UME6* consequently enables filamentation of *C. dubliniensis*. Interestingly, Ume6 interacts with the chromatin remodeling complex Isw2 in *S. cerevisiae* to repress expression of meiotic genes (Goldmark et al., 2000) and a *C. albicans isw2*Δ mutant fails to form chlamydospores (Nobile et al., 2003). Hence, we tested sporulation of a *C. albicans ume6*Δ deletion as well as a *UME6*^*OE*^ overexpression strain on standard and peptone-supplemented CM agar. Against our expectation, the *ume6*Δ mutant was not impaired in chlamydosporulation on CM agar, and *UME6* overexpression did not allow chlamydospore formation on peptone- or glucose-containing media, although hyphae were formed readily (Figure S2). In contrast, deletion of the filamentation repressor *NRG1* in *C. albicans* was epistatic to the glucose- or peptone-induced chlamydospore inhibition and its effect could only be reversed by ammonium sulfate or high peptone levels (Table 2). Therefore, we concluded that a strong link between chlamydospore and filamentation regulation exists, but that *UME6* is not a key factor in the former.

### Nitrogen-sensing pathways promote chlamydospore formation

**Figure 2:**
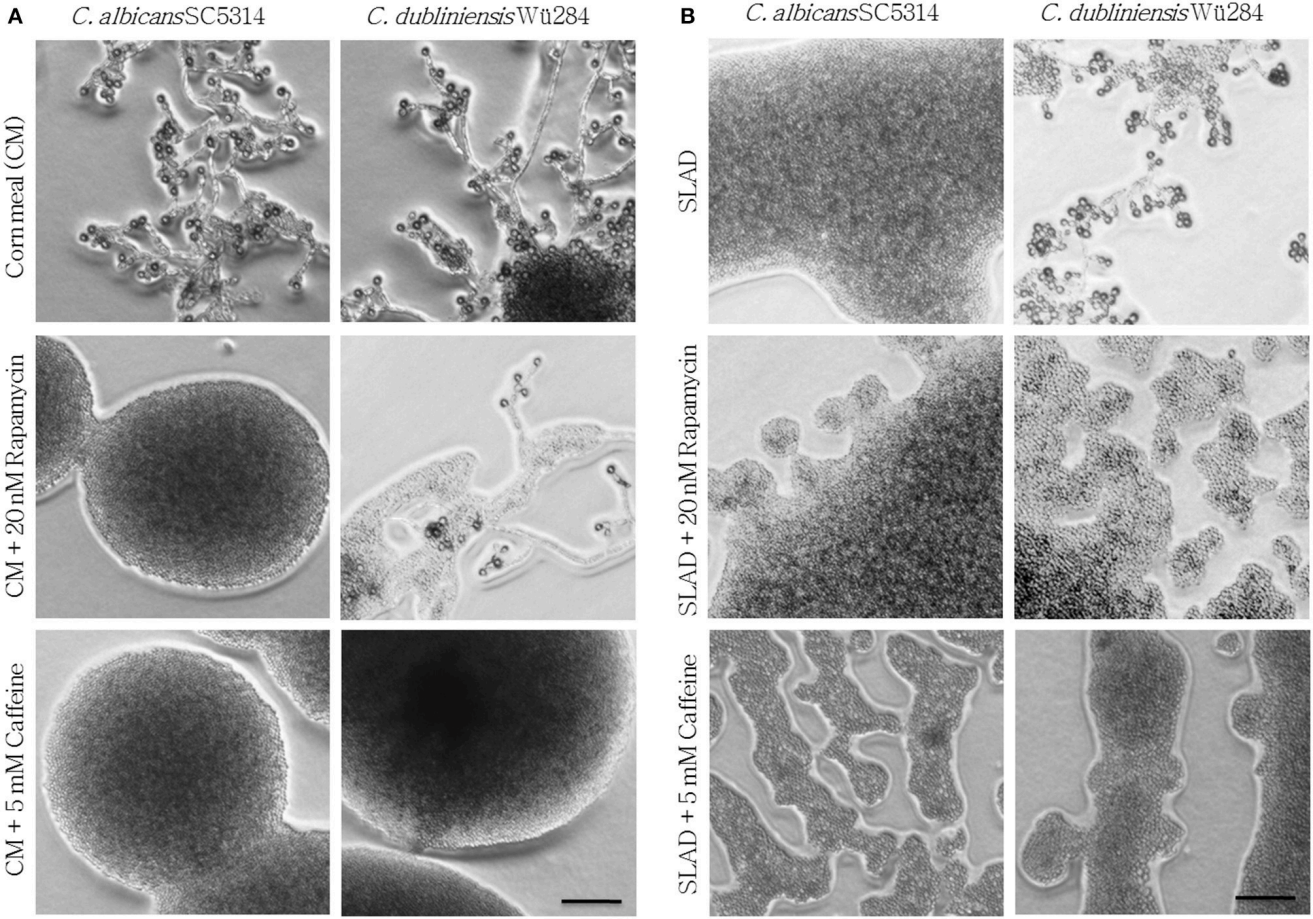
**Tor antagonists inhibit chlamydospore formation of ***C. albicans*** and ***C. dubliniensis*****. *Candida* wild type strains were streaked onto **(A)** CM or **(B)** SLAD (YNB + 2% glucose) agar with added TOR antagonists. On day 7 of incubation (27°C, darkness), the colony morphology showed that inhibition of TOR signaling led to a significant repression of sporulation (CI 0–1). Scale bars, 100 μm.

Eukaryotes sense availability of nutrients like nitrogen and glucose via the highly conserved Tor1 kinase complex. Under conditions of sufficient nutrient supply, Tor1 is active and drives a signal cascade that supports cell proliferation, whereas Tor1 inhibition under starvation mediates autophagy (Kamada et al., 2004) and stimulates nitrogen uptake as well as expression of nitrogen catabolite-repressed genes (Cardenas et al., 1999). We hence supplemented chlamydospore-inducing media with the Tor1 antagonists rapamycin and caffeine to investigate a possible role of Tor1-signaling in sporulation. Sublethal rapamycin (20 nM) and caffeine (5 mM) concentrations allowed sustained growth, but efficiently abolished chlamydospore formation of *C. albicans* on CM agar (Figure 2A). Similarly, chlamydospore formation was also strongly reduced in *C. dubliniensis*, although some sporulation remained (CI 1) even under rapamycin treatment (Figure 2A). To further investigate this inhibition, we next tested the response to rapamycin and caffeine on the *C. dubliniensis*-specific chlamydospore inducer medium, SLAD agar (YNB + 2% glucose). Here both substances totally abolished sporulation (Figure 2B), and therefore rapamycin had an even more severe effect on *C. dubliniensis* than in CM agar-based assays. Similar effects were seen on chlamydospore-inducing niger seed (*Guizotia abyssinica*) agar (Staib and Morschhäuser, 1999) or rice agar (data not shown) which suggests a conserved TOR-related mechanism independent of the specific inducing medium.

How can the nutrient sensor TOR influence fungal morphology on low-nitrogen agar? Under nitrogen starvation, diploid *S. cerevisiae* cells are known to grow as pseudohyphae that invade SLAD agar surfaces (Gimeno et al., 1992), and this morphological transition can be abolished by rapamycin treatment (Cutler et al., 2001). Therefore, it has been suggested that the pseudohyphae of baker's yeast rely on a low, but stable TOR activity during nitrogen starvation (Cutler et al., 2001). According to this model, both the presence of nutrients with its increased TOR activity as well as rapamycin-mediated repression of the TOR pathway can lead to inhibition of filamentation (Cutler et al., 2001). Parallels can be drawn to our observation of a rapamycin-sensitive chlamydospore formation by *Candida* spp., and we thus propose that similar to *S. cerevisiae* pseudohyphae, a limited amount of Tor1 activity is needed to induce chlamydospores. Differences in basal TOR activity levels and distinct thresholds to supplementary nutrients and Tor1 antagonists could thus explain the species-specific response.

Analogous to pseudohyphal growth of *S. cerevisiae*, addition of TOR inhibitors to SLAD agar also blocks hyphal growth of *C. albicans* and pseudohyphal growth of *Candida lusitaniae* and *Cryptococcus neoformans* (Cutler et al., 2001). Therefore, it seems that chlamydospore formation relies on conserved morphogenesis pathways that are dependent on the nutritional state sensor TOR, but adapted to drive different growth forms during evolution.

As *C. albicans nrg1*Δ sporulated nearly constitutively even in presence of several good nutrient sources, the mutant strain was similarly treated with rapamycin and caffeine. However, even in the presence of inhibitors chlamydospore formation was not reduced (Figure S3), although the colonies appeared smaller. A likely explanation is that nutrient-driven pathways, including the TOR network, positively control the transcriptional repressor Nrg1. In line with this suggestion, gene expression of *NRG1* was shown to be higher in *C. albicans* than *C. dubliniensis* under conditions where chlamydospore formation was observed only in the latter (Staib and Morschhäuser, 2005). In combination with our data, this suggests that a low but stable TOR activity keeps *NRG1* expression at such reduced levels that allow the intense chlamydosporulation of *C. dubliniensis*. Analogous observations have been made during hyphae development, were both, rapamycin treatment and *NRG1* deletion, in *C. dubliniensis* phenocopied *C. albicans*-like filamentation in liquid media (Sullivan and Moran, 2011).

### Tor1 antagonists directly impact chlamydosporulation and associated gene expression

**Figure 3:**
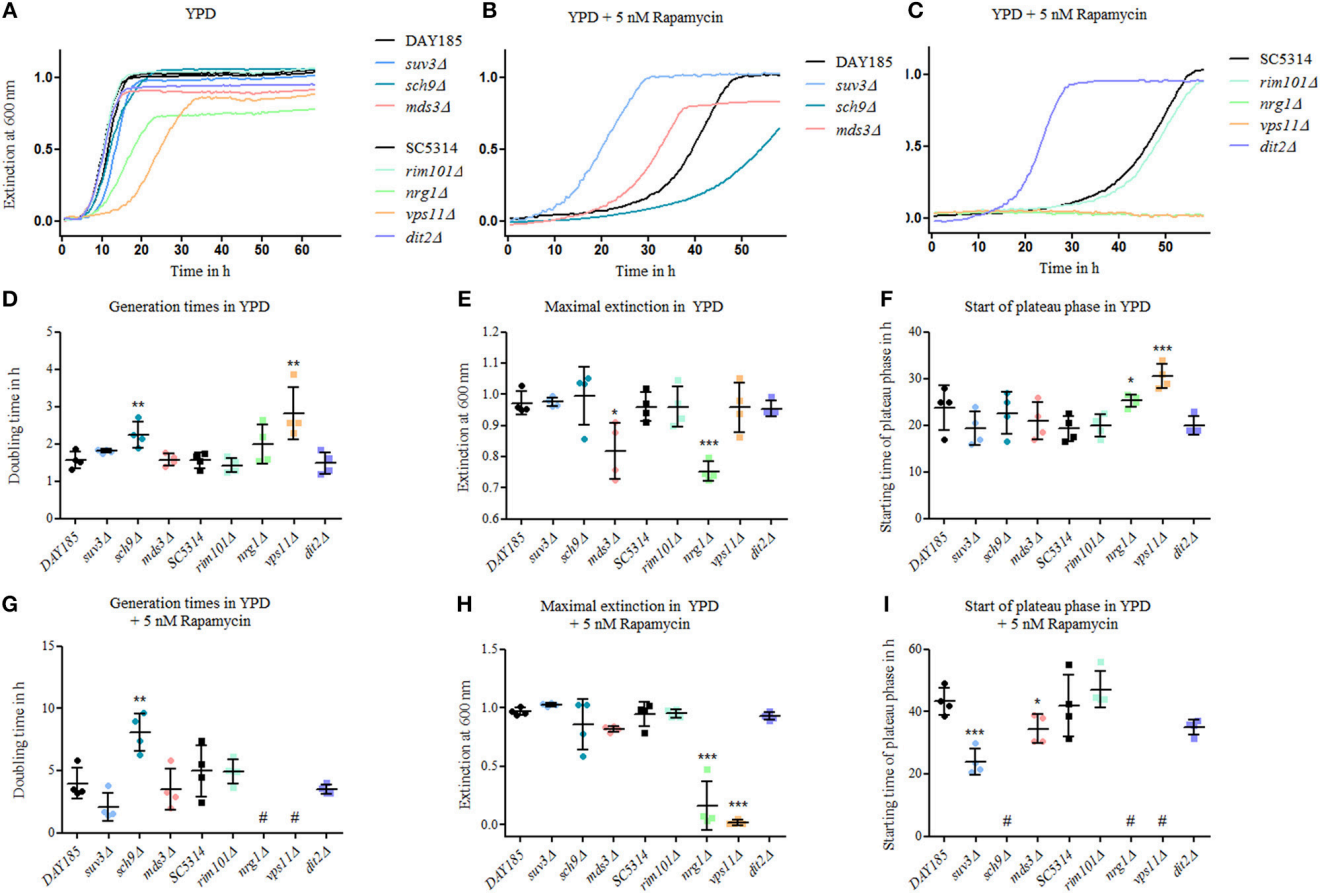
**Mutants of chlamydospore-associated ***C. albicans*** genes are resistant to rapamycin**. Growth of *Candida* strains (*C. albicans* parental strains SC5314 and DAY185 and *C. albicans* deletion mutants) from an overnight YPD preculture was monitored in **(A)** YPD, **(B)** and **(C)** in YPD plus 5 nM rapamycin and exemplary growth curves are shown. Several of the mutants showed an altered—mainly increased—rapamycin resistance. Mutant strains are grouped according to their relevant parental strain, DAY185 **(B)** or SC5314 **(C)**. Growth rates were analyzed using scatter plots with means and SD-values by **(D)** and **(G)** generation times in log phases, **(E)** and **(H)** maximum extinction values at plateau phases and **(F)** and **(I)** time for reaching plateau phases, and statistical analyses of mutant strains were performed in comparison to their parental strains (*n* = 4, # no growth data for analyses, statistics: One-way analysis of variance [ANOVA], *post-hoc* test with Tukey multiple testing correction for *P* < 0.05, significance levels compared to control: ^***^p < 0.001; ^**^p < 0.01; ^*^p < 0.05).

Currently, 19 genes are known to be essential for proper chlamydosporulation in *C. albicans*, among them two genes encoding key components of the TOR regulatory pathway: Sch9 and Mds3 (Sonneborn et al., 1999; Davis et al., 2002; Nobile et al., 2003; Staib and Morschhäuser, 2005; Eisman et al., 2006). To follow up on the connection between TOR signaling and chlamydospore formation, we tested the resistance and growth of known chlamydospore-defective *C. albicans* mutants (*suv3*Δ, *sch9*Δ, *mds3*Δ, *rim101*Δ, *vps11*Δ, *dit2*Δ) as well as of the hypersporulative *nrg1*Δ mutant, in the presence of rapamycin as altered rapamycin resistance can serve as an indicator for a role in the TOR signaling cascade (Huang et al., 2004). All mutants were viable and most grew at roughly wild-type levels in YPD without drug, with slightly, but significantly prolonged generation times of the *sch9*Δ and *vps11*Δ mutant strains (Figures 3A,D). The *nrg1*Δ and the *mds3*Δ strains showed a more severe reduction in optical density (Figure 3E) due to and in proportion to their hyperfilamentous morphology. Moreover, *nrg1*Δ and *vps11*Δ reached stationary growth phases later than the other strains (Figures 3A,F). In presence of rapamycin, we identified three groups of mutants: The strongly resistant strains *suv3*Δ*, mds3*Δ, and *dit2*Δ with early stationary phases (Figure 3I); the highly susceptible strains *sch9*Δ*, vps11*Δ, and *nrg1*Δ showing significantly increased doubling times or even no growth (Figures 3G–I); and the strain *rim101*Δ, the growth of which was not influenced by rapamycin. These results mirror the key role of TOR signaling during chlamydospore formation, but additional TOR-independent pathways seem to exist, as indicated by the rapamycin-unaffected mutant lacking the transcription factor Rim101. A possible explanation may be that chlamydospore-inducing media generally have a slightly acidic pH around 5–6 (Montazeri and Hedrick, 1984), and pH-signaling is mediated by Rim101 and its activator Rim13, both of which are known to be essential for full chlamydosporulation (Nobile et al., 2003).

### Key factors of the tor signaling cascade are required in chlamydospore production

**Figure 4:**
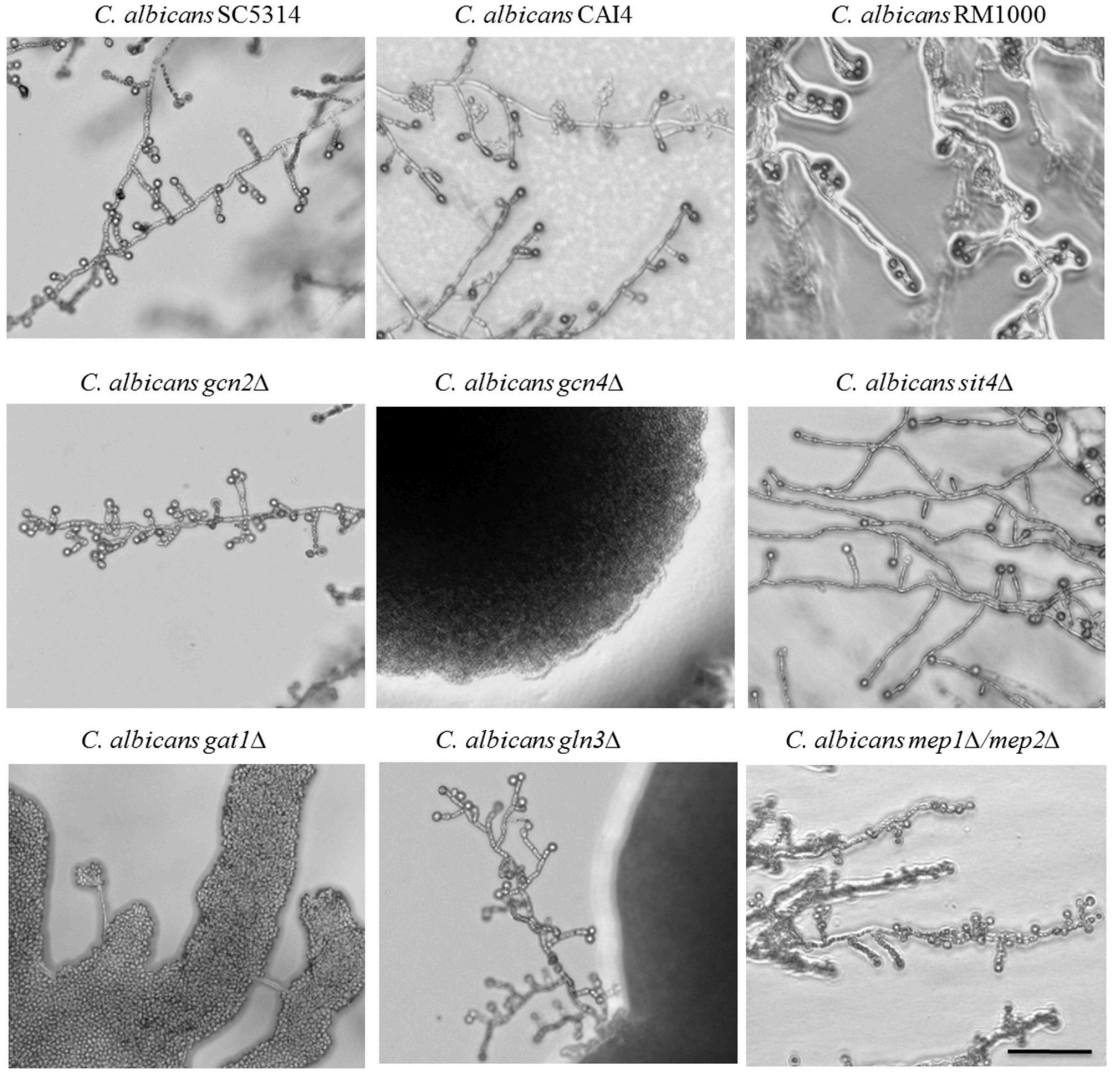
**Identification of chlamydospore-defective ***C. albicans*** TOR pathway mutants**. *C. albicans* parental (SC5314, CAI4, RM1000) and deletion strains were grown 7 days at 27°C in darkness on CM agar plates. Several of the strains defective in TOR pathway components exhibited abolished chlamydospore formation: the *gcn4*Δ and *gat1*Δ mutants failed chlamydosporulation (CI 0) and the *gln3*Δ mutant was only capable to produce few chlamydospores (CI 1) with a time delay. Scale bar, 100 μm.

Our analyses thus showed a connection to TOR signaling, which comprises a complex network that controls growth and longevity in eukaryotes as well as morphology in fungi (Shertz and Cardenas, 2011). In order to better understand the function of this signaling cascade during chlamydospore formation, we went on to screen a selection of *C. albicans* TOR signaling deletion mutants for chlamydospore production on CM agar (Figure 4). A key target of the Tor complex is the Sit4 phosphatase, which has multiple cellular functions including regulation of cell cycle, cytoskeleton assembly, and rDNA gene expression (Angeles de la Torre-Ruiz et al., 2002). The loss of Sit4 in *S. cerevisiae* results in defective pseudohyphal differentiation (Cutler et al., 2001), and a *C. albicans sit4*Δ mutant is both afilamentous and avirulent (Lee et al., 2004). In contrast, on CM agar a *C. albicans sit4*Δ deletion strain showed no defect in chlamydosporulation (Figure 4), indicating no function of Sit4 during chlamydospore formation, in stark contrast to filamentation.

In the presence of sufficient nutrients, the TOR pathway blocks uptake and catabolism of nitrogenous substrates, but under nitrogen-poor conditions, it facilitates nuclear entry of the GATA factors Gat1 and Gln3 to mediate expression of nitrogen catabolite repressed (NCR) genes (Beck and Hall, 1999). In parallel, TOR represses the expression of amino acid biosynthesis genes under sufficient nutrient availability via the eIF2α kinase Gcn2 that activates the transcription factor Gcn4. Thus, regulation occurs on transcriptional, translational and post-translational levels (Cherkasova and Hinnebusch, 2003). The role of these two nitrogen-responsive cascades for chlamydospore formation was assayed using *C. albicans* deletion mutants of key factors on CM agar.

Both pathways seem to play an important role during chlamydospore development, because deletion of either the central activator of amino acid biosynthetic genes, Gcn4 (Tripathi et al., 2002), or the transcriptional activator of NCR genes, Gat1 (Limjindaporn et al., 2003), caused chlamydospore deficiency (Figure 4). In fact, the *C. albicans gat1*Δ deletion mutant is a prime example for distinct chlamydospore and hypha regulation programs, as the chlamydospore defect was independent of its filamentation capacity. In contrast to our results, a previous study on the role of *C. albicans* Gat1 during morphogenesis found no reduction in both, hyphae and chlamydospore formation (Limjindaporn et al., 2003), possibly due to media-specific differences. Overall, a diminished TOR activity and the resultant starvation response thus seem to mediate chlamydospore formation.

The second regulator of NCR gene expression is the transcription factor Gln3, which shares many functions with Gat1 (Liao et al., 2008). Deletion of *GLN3* resulted in a reduced efficiency of chlamydospore formation combined with an extended sporulation initiation. Both GATA factors, Gln3 and Gat1, enable yeasts to selectively utilize preferential nitrogen sources via NCR, a mechanism which is indispensible for fungal pathogens like *Aspergillus fumigatus* and *C. albicans* during infections (Lee et al., 2013). Of about 100 direct NCR target genes we hence chose to investigate the role of the ammonium transporter genes, encoding Mep1 and Mep2, by testing the corresponding mutants for their chlamydosporulation capability. To exclude an effect of the functional redundancy of these transporters, a *C. albicans mep1*Δ*/mep2*Δ double knockout mutant (Biswas and Morschhäuser, 2005) was used. Sporulation was unimpaired in this mutant, in agreement with the non-repressive effect of additional ammonium in CM agar. Thus, this ammonium-specific pathway seems not to be involved in sensing a chlamydospore-inducing environment. This phenotype stands in an interesting contrast to the impact of the high affinity transporter Mep2 on hyphae formation: The N-terminal region of the transporter is essential for *C. albicans* filamentation on SLAD agar (Biswas and Morschhäuser, 2005).

In contrast to ammonium, amino acid homeostasis is under control of Gcn2, which activates Gcn4—a pathway that is controlled by TOR under amino acid excess (Tournu et al., 2005). Whereas, Gcn4 is known to be essential under amino acid starvation and to have a large impact on yeast-to-hypha transitions in *C. albicans* (Tripathi et al., 2002), Gcn2 has a minor role in filamentation processes and is only involved in N-acetylglucosamine (GlcNAc)-induced hyphae development (Tournu et al., 2005; Kamthan et al., 2012). In our experiments, the deletion of *GCN4*, but not *GCN2*, inhibited chlamydospore formation. This suggests a Gcn2-independent route of Gcn4 activation under our tested conditions. In agreement with our previously observed peptone-induced repression, the amino acid starvation response seems to be a crucial element during chlamydospore development by *C. albicans*.

### Fermentable carbon sources specifically inhibit chlamydospore formation by *C. albicans*

**Figure 5:**
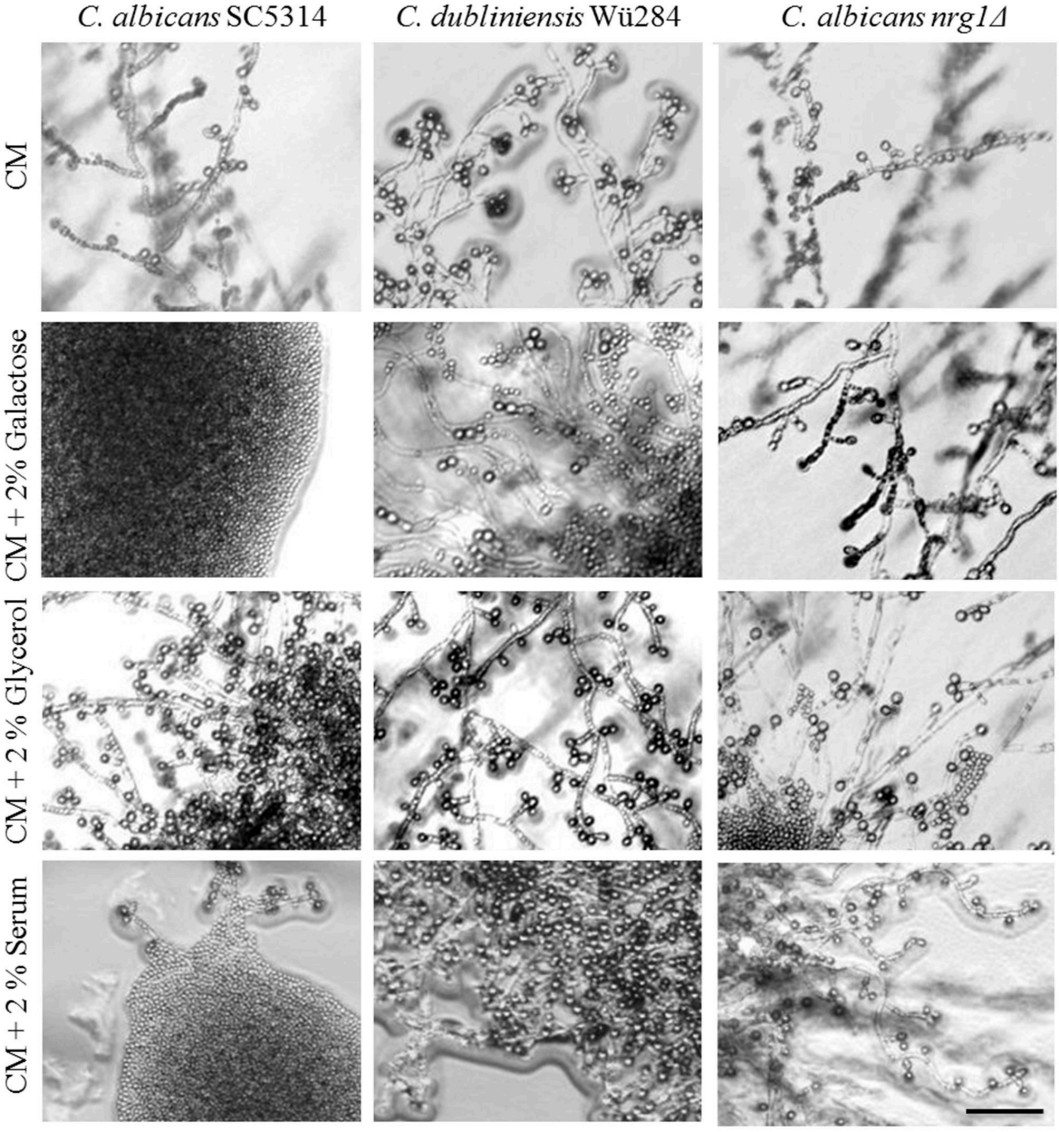
**Effect of carbon sources on chlamydospore formation**. *Candida* strains were streaked on pure or supplemented CM agar and grown 7 days at 27°C in darkness. The addition of fermentable sugars like galactose or glucose-rich serum inhibited chlamydospore formation species-specifically in *C. albicans* (CI 0–1), but the *NRG1* gene deletion reversed this phenotype (CI 3). Scale bar, 100 μm.

Common chlamydospore-inducing media are based on corn or rice and are therefore rich in starch, indicating that polysaccharides have an inducing or at least non-repressing effect on chlamydospore development. Complex carbohydrates are known to influence fungal morphology: In *S. cerevisiae*, starch degradation is associated with the ability to grow invasively and form pseudohyphae (Vivier et al., 1997) and polymeric cyclodextrines were described to induce hyphae by *C. albicans* (Fekete-Forgács et al., 1997). The repressing effect of glucose on chlamydosporulation, in contrast, was described almost half a century ago for *C. albicans* (Jansons and Nickerson, 1970b). Here, we tested a variety of carbon sources supplemented to CM and YBN agars (Table 2 and Figure 5) and analyzed their effect on *C. albicans* and *C. dubliniensis* wild type strains as well as the *C. albicans nrg1*Δ mutant. A direct correlation of the presence of fermentable mono- and disaccharides (glucose, galactose, maltose, sucrose, and glucose-containing serum) and inhibition of chlamydospore development was evident only for *C. albicans*. Furthermore, glucose is known to be the dialysable germ tube-inducing component in serum (Hudson et al., 2004) and the addition of 2% serum to CM agar similarly blocked chlamydospore formation by *C. albicans*. Hence, both glucose and serum, seem to play opposing roles during hyphae formation on one side and chlamydospore development on the other. In contrast, chlamydospore formation of both, the *C. dubliniensis* wild type strain and the *C. albicans nrg1*Δ mutant, was resistant to the addition of sugars. Similar differences exist in filamentation: Serum alone is not sufficient to induce hyphae by *C. dubliniensis* and additional glucose inhibits hyphae formation to some extent (O'Connor et al., 2010). Together, these results point toward a rewired glucose response under chlamydospore-inducing conditions: Glucose is an important hyphae inducer and chlamydospore repressor of *C. albicans*, whereas the same substrate plays the opposite, albeit less pronounced role in morphogenic transitions of *C. dubliniensis*. Our results with the *C. albicans nrg1*Δ mutant furthermore indicate that the nutrient-induced chlamydospore repression of *C. albicans* is likely due to Nrg1-mediated repression of gene expression. A glucose-driven regulation of Nrg1 has been described in the fungal pathogen *C. neoformans*, where expression of the *NRG1* homolog is controlled in response to carbohydrate availability (Cramer et al., 2006).

Notably, supplementation with non-fermentable carbon sources like glycerol and lactose did not inhibit, but rather enhanced sporulation by *C. albicans* (Figure 5), suggesting that only readily utilizable nutrients can block the chlamydospore formation in response to starvation. We found similar repressing effects by both glucose and peptone for *C. albicans*, but in other fungi saccharides have a significantly higher inhibitory potential than nitrogen sources. A low carbon-nitrogen ratio is, for example, the most important inductor of chlamydospore production in soil-borne *Fusarium* species (Griffin, 1976), where they serve as dormant structures under starvation conditions (Smolinska, 2000).

### cAMP signaling mediates activation of chlamydospore-associated transcription factors

Glucose has a broad range of effects on morphogenetic switches, and, in yeasts, its downstream signaling is mediated mainly via the Ras-cAMP-protein kinase A (PKA) pathway (Feng et al., 1999; Hudson et al., 2004). TOR signaling plays also a minor role in the glucose response (Hardwick et al., 1999). Overlaps exist, e.g., in form of Tor1 effector kinase Sch9 which inhibits PKA activity (Zhang et al., 2011) and as a suppression of TOR deficiency due to constitutive stimulation of the RAS-cAMP pathway in yeast (Schmelzle et al., 2004). Therefore, a reliable association of one nutritional trigger or repressor of chlamydospore formation to a distinct pathway is difficult. We therefore investigated the possible role of the Ras-cAMP-PKA pathway during chlamydosporulation directly. A first link toward cAMP signaling was indicated by a study on the transcriptional activator Efg1. In *C. albicans*, Efg1 acts as a downstream effector of the cAMP-PKA pathway, and an *efg1*Δ deletion mutant exhibited a chlamydosporulation defect (Sonneborn et al., 1999). We thus systematically investigated more pathway members for their function in chlamydospore formation.

Different environmental stimuli, including hyphae-initiating conditions, activate the Ras1 GTPase. This initiates a cascade by the activation of the Cyr1 adenylyl cyclase, which catalyzes the formation of cyclic AMP (cAMP) (Rocha et al., 2001). High levels of cAMP in turn activate PKA, which phosphorylates and thus regulates transcription factors like Efg1 (Inglis and Sherlock, 2013). The level of cAMP is tightly regulated in response to external stimuli, and cAMP hydrolysis is driven by the high affinity phosphodiesterase Pde2 (see also **Figure 8**).

**Figure 6:**
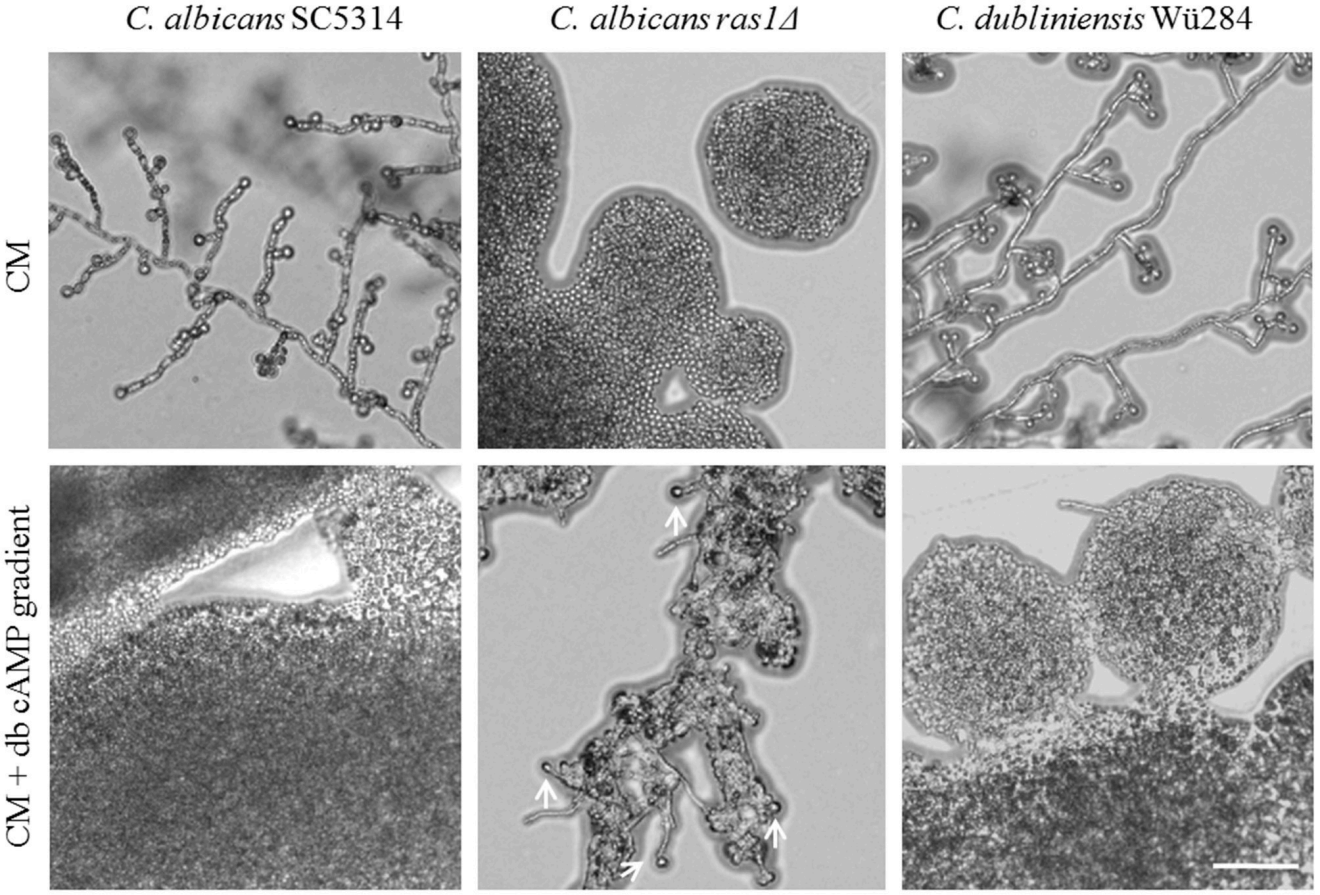
**Role of cAMP signaling during chlamydospore formation**. *Candida* strains were grown on CM agar with or without a cAMP gradient. At high cAMP concentrations chlamydospore formation was inhibited in *C. albicans* and *C. dubliniensis* wild type strains (CI 0). In contrast, the *C. albicans ras1*Δ mutant did not show chlamydospore formation on CM medium (CI 0), but this was partially restored by addition of cAMP (white arrows indicate chlamydospores formed by the *C. albicans ras1*Δ mutant, CI 1). Scale bar, 100 μm.

A mutant lacking *RAS1* in *C. albicans* lost the ability to form chlamydospores (Figure 6). The *ras1*Δ mutant is also known to be afilamentous on several media, but hyphae formation can be regained by supplementation with cell-permeable dibutyryl (db-) cAMP (Rocha et al., 2001). To test its effect on sporulation, we produced CM agar plates with a concentration gradient of db-cAMP by punching a hole into the agar and filling it with a solution of 100 mM db-cAMP. High db-cAMP concentrations fully inhibited sporulation of both *Candida* wild type strains alike. However, at the same concentrations, we identified a zone of increased chlamydospore formation for the *C. albicans ras1*Δ mutant (Figure 6). A similar effect was observed using a *cyr1*Δ mutant (not shown). In further experiments, we found that a concentration of 10 mM db-cAMP in CM agar was sufficient to inhibit chlamydospores formation in the wild type, but induced sporulation in the *ras1*Δ mutant (data not shown). Moreover, addition of 10 mM db-cAMP inhibited sporulation of *C. dubliniensis* on SLAD agar. Reasoning that, based on the *ras1*Δ phenotype, a certain level of cAMP is required for chlamydosporulation, we tested additional db-cAMP under repressive conditions like nutrient-rich CM agar. However, we were not able to re-induce chlamydospores by *C. albicans* or *C. dubliniensis* (Figure S4).

The intracellular level of cAMP is tightly regulated and the Pde2 phosphodiesterase degrades cAMP (Jung and Stateva, 2003). We thus used a *C. albicans pde2*Δ deletion mutant to investigate the effect of cAMP overload on chlamydosporulation. As expected, on CM agar a *C. albicans pde2*Δ deletion mutant produced significantly more hyphae than the wild type (Jung and Stateva, 2003), but, interestingly, chlamydospore formation was normal (Figure S5). Similarly, it has previously been shown that heterologous expression of *CaPDE2* in *C. dubliniensis* reduced filamentation, but did not modify chlamydospore production on the diagnostic niger (*G. abyssinica*) agar (Staib and Morschhäuser, 2005). In contrast to the *nrg1*Δ mutant, *PDE2* deletion did not rescue sporulation in presence of rapamycin or glucose on CM agar (Figure S5). Therefore, the *C. albicans pde2*Δ mutant is another example where hyphae and chlamydospore formations are distinctly regulated.

Active RAS-cAMP signaling was thus shown to be essential to initiate chlamydospores, but no significant species-specific differences were evident. This is reminiscent of the fact that cAMP levels are not at the basis of the characteristically low hyphae formation of *C. dubliniensis*, and that neither a hyperactive Ras1 nor elevated cAMP levels can enhance its filamentation (Moran et al., 2007). Supporting this notion, comparative genomics revealed that the *C. albicans* and *C. dubliniensis* Ras-cAMP pathway are not diverged (Jackson et al., 2009).

### Chlamydospore repression by light is specific for *C. albicans*

**Figure 7:**
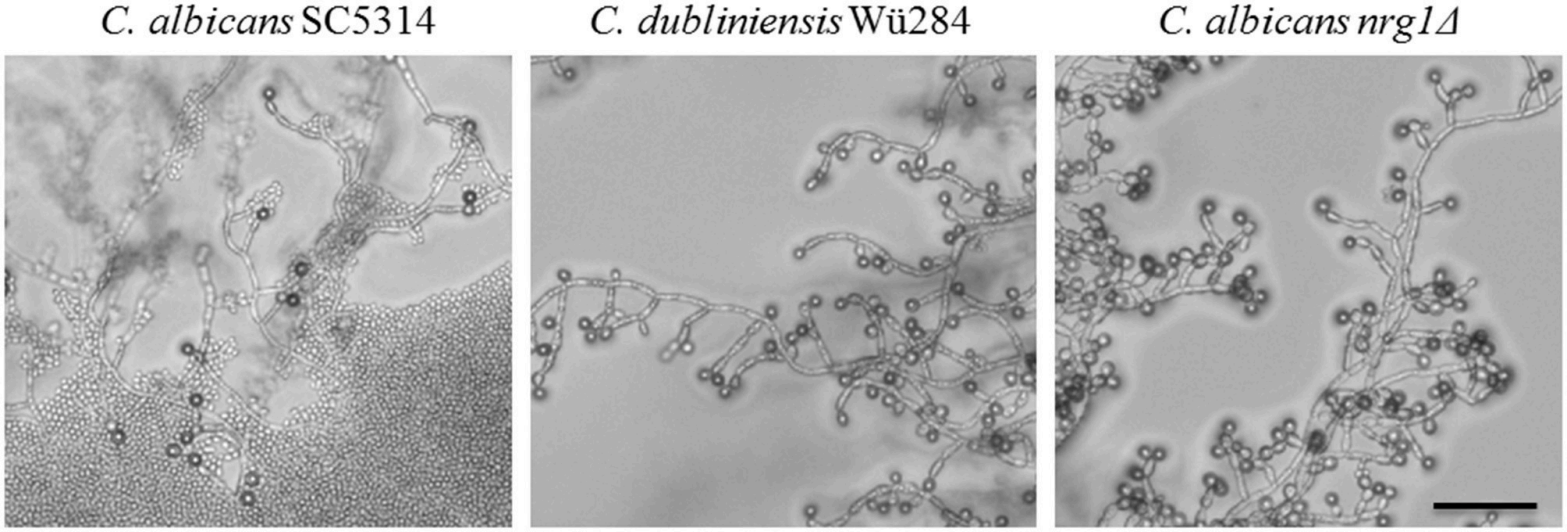
**Light exposure reduces chlamydosporulation specifically of ***C. albicans*****. *Candida* strains were grown on CM agar at 27°C for 7 days under permanent light. The number of chlamydospores formed by the *C. albicans* wild type was abolished in areas with low cell density and high light exposures (CI 0). Only in zones with higher cell densities a limited chlamydospore formation was visible (CI 1). In contrast, the *C. dubliniensis* wild type and the *C. albicans nrg1*Δ strain formed chlamydospores at levels comparable to incubation in the dark (CI 3). Scale bar, 100 μm.

Finally, we tested the effect of light on chlamydosporulation in *Candida*. Incubation in darkness has been known for a long time as an inducer of chlamydospore formation by *C. albicans* (Dujardin et al., 1980a), but the light-sensing mechanism or its regulators are still unidentified. We verified the light sensitivity of the *C. albicans* wild type strain, which mostly failed chlamydosporulation on CM upon permanent light exposure. Interestingly, although complete darkness is generally thought to be required for their production, we observed *C. albicans* chlamydospores in areas of high cell density even under light (Figure 7). Furthermore, in contrast to the *C. albicans* wild type, both *C. dubliniensis* and the *C. albicans nrg1*Δ deletion mutant readily produced unaltered (CI 3) amounts of chlamydospores regardless of light exposure and cell density (Figure 7). The restoration of chlamydosporulation by the *C. albicans nrg1*Δ strain suggests that light exposure may be signaled via a pathway that triggers the central repressor Nrg1. On the other hand, *C. dubliniensis* is often found in environmental samples (Nunn et al., 2007; McManus et al., 2009), which may have selected for a niche-adapted higher resistance to sunlight.

Other fungal species use light as a signal to regulate their life cycle and metabolism [reviewed in (Tisch and Schmoll, 2010)]. In the filamentous fungus *A. nidulans*, for example, light is sensed via the velvet complex, which controls sexual reproduction and production of secondary metabolites (Bayram et al., 2008). Velvet complex mutants of *A. nidulans* consequently fail conidiation and sexual sporulation (Kato et al., 2003). In addition, light sensing controls glucose metabolism in filamentous fungi, as illumination influences glycolysis, the pentose phosphate pathway, or the Krebs cycle (Tisch and Schmoll, 2010). All these processes are involved in glucose starvation and could therefore provide a link to the regulation of chlamydospore production in *Candida*. Comparative protein sequence analysis (PBLAST), however, revealed no evident homologs of phytochromes or other light sensors in *Candida* species. Therefore, although we found some clues to the signal transduction and the species-specific differences, the mechanism of light sensing itself remains obscure. Given the absence of evident light sensors, production of reactive oxygen species during light exposure may be suggested as an alternative mechanism, which could feasibly connect light to the chlamydospore-negative phenotypes of the *C. albicans hog1*Δ and *goa1*Δ deletion mutants, as the deleted genes are involved in stress responses (Eisman et al., 2006; Bambach et al., 2009).

## Conclusion

**Figure 8:**
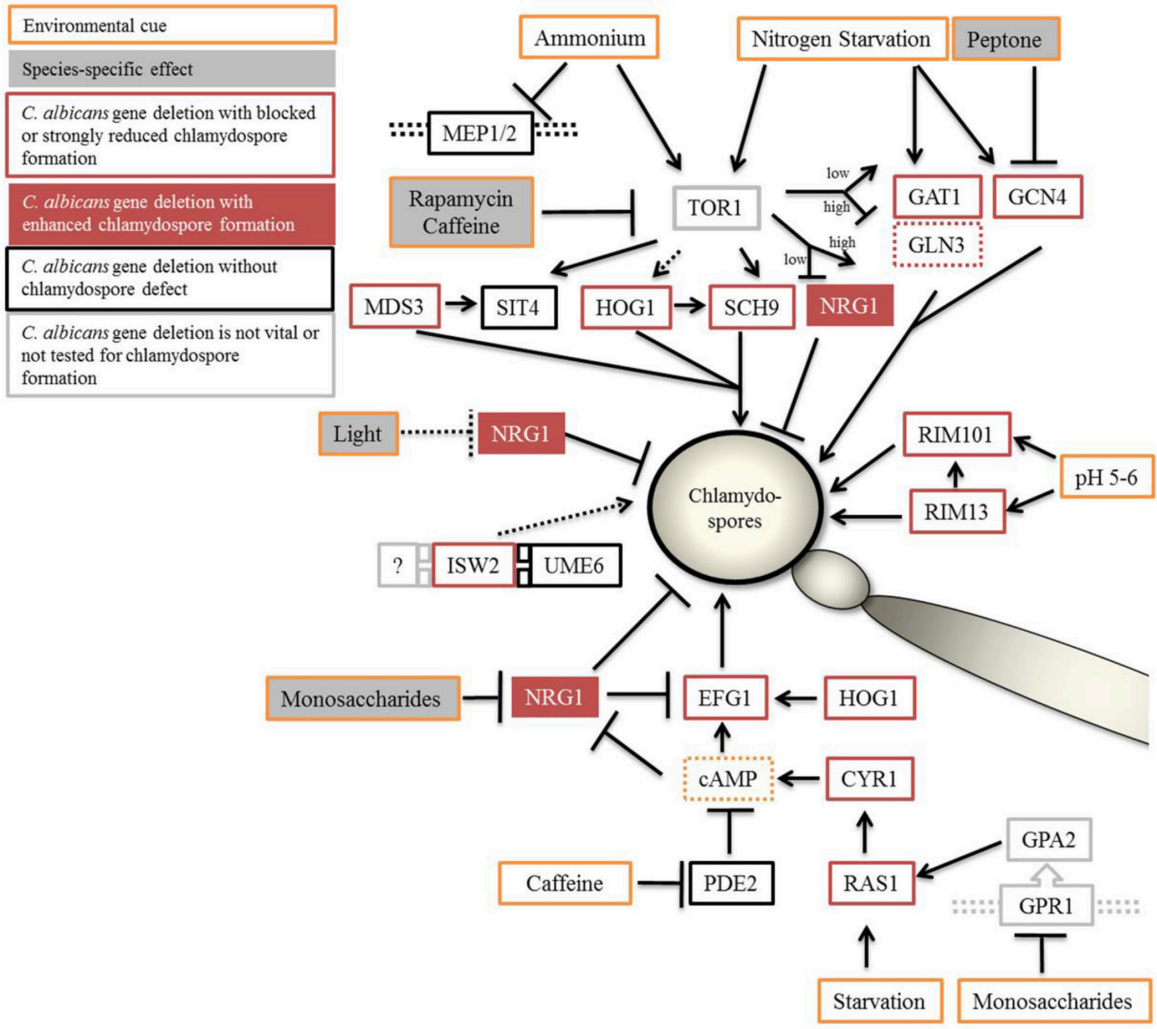
**Proposed model of chlamydospore regulation in ***Candida*** species**. Optimal conditions of chlamydospore formation by *C. albicans* and *C. dubliniensis* are room temperature (27°C), darkness, and poor nutritional conditions (although the presence of starch-containing plant material has a supporting effect). The TOR and cAMP-PKA signaling pathways relay the environmental status during chlamydospore initiation and signaling. Both pathways can be suppressed by the addition of nutrients, especially fermentable saccharides and peptone, with *C. dubliniensis* tolerating higher concentrations of such nutrients. In *C. albicans, NRG1* was found epistatic to nutrient supplementation and seems to be the main negative regulator in response to nutrients. Direct inhibition of the Tor1 kinase complex led to stark reduction in chlamydosporulation in both species. Light radiation had a *NRG1*-dependent repressing effect only on *C. albicans*. Orange outline, inducing and repressing environmental factors; gray filling, species-dependent effects. Black outline, *C. albicans* gene deletions were chlamydospore-positive, or red outline, -negative on CM agar. Red filling, *NRG1* to mark the hypersporulative phenotype of its deletion mutants.

Although chlamydospores of *C. albicans* have been under investigation for several decades, knowledge on their induction and regulation pathways is still fragmentary. On the other hand, assays which are based on the species-specific chlamydospore initiation are a widespread diagnostic method to distinguish between *C. albicans* and *C. dubliniensis* (Ells et al., 2011). In this comparative study, we have examined chlamydospore-inducing signaling cascades in response to nutrients availability in both species and found important differences. The *C. albicans*-specific and Nrg1-dependent suppressive effect of several monosaccharides and peptone indicate that differing Nrg1 activity levels is likely one key to the species-specific chlamydospore formation on diverse media (this study and Staib and Morschhäuser, 2005). Our data on chlamydospore-negative *C. albicans* deletion mutants as well as the TOR and cAMP-signaling mutants revealed that chlamydospore formation relies mainly on the TOR and Ras-cAMP-PKA signaling cascades (Figure 8). As the TOR pathway relays on nutritional signals, and inhibition of Tor1 kinase activity prevented sporulation to a lower degree in *C. dubliniensis*, our hypothesis is that *C. dubliniensis* has intrinsically elevated levels of Tor1 activity which allow chlamydospore formation under conditions that are not sufficient to induce a starvation response in *C. albicans*. The chlamydosporulation defect of *C. albicans ras1*Δ was rescued by supplementation with cAMP, hinting toward a minimum requirement of cAMP, while a surplus of cAMP negatively affects chlamydospore formation of both *Candida* species, implying the presence of an optimal cAMP range for sporulation initiation.

Light, a common factor in the control of formation of sexual fungal spores, had a specific effect on *C. albicans* chlamydospore formation, which was reversible by *NRG1* gene deletion. As light exposure had an effect similar to nutrient supplementation, including the signaling via Nrg1, an overlap between these signaling pathways seems likely, although the topic of (species-specific) light sensing remains an important unexplored topic in chlamydosporulation.

In summary, this work expands our knowledge of chlamydospore formation by *Candida* species as a morphological transition next to the conversions between yeasts, pseudohyphae, and hyphae. We found that chlamydosporulation is a complex process that acts via and integrates different signal transduction pathways. While overlaps with hyphae-inducing pathways were obvious, some major hyphae-regulating factors like Ume6, Mep2, and Pde2 play only a minor role during sporulation, suggesting network re-use and rewiring during the evolution of this specific morphogenetic process.

## Author contributions

Conceived and designed the experiments: BB, PS, SB. Performed the experiments: BB, CP. Analyzed the data: BB, CP, BH, SB. Wrote the paper: BB, SB, BH.

## Funding

This study was supported by Deutsche Forschungsgemeinschaft (DFG) grant STA 1147/1-1 and by the German Federal Ministry of Education and Health (BMBF) Germany (FKZ: 01EO1002, Integrated Research and Treatment Center, Center for Sepsis Control and Care (CSCC).

### Conflict of interest statement

The authors declare that the research was conducted in the absence of any commercial or financial relationships that could be construed as a potential conflict of interest.
