# Supplementary material for: Candida species Rewired Hyphae Developmental Programs for Chlamydospore Formation
Source: Front Microbiol. 2016 Oct 27;7:1697. doi: 10.3389/fmicb.2016.01697 (PMC5081361; doi:10.3389/fmicb.2016.01697)
Supplement: Supplementary file 1 [file DataSheet1.DOCX]

# Supplementary materials

**
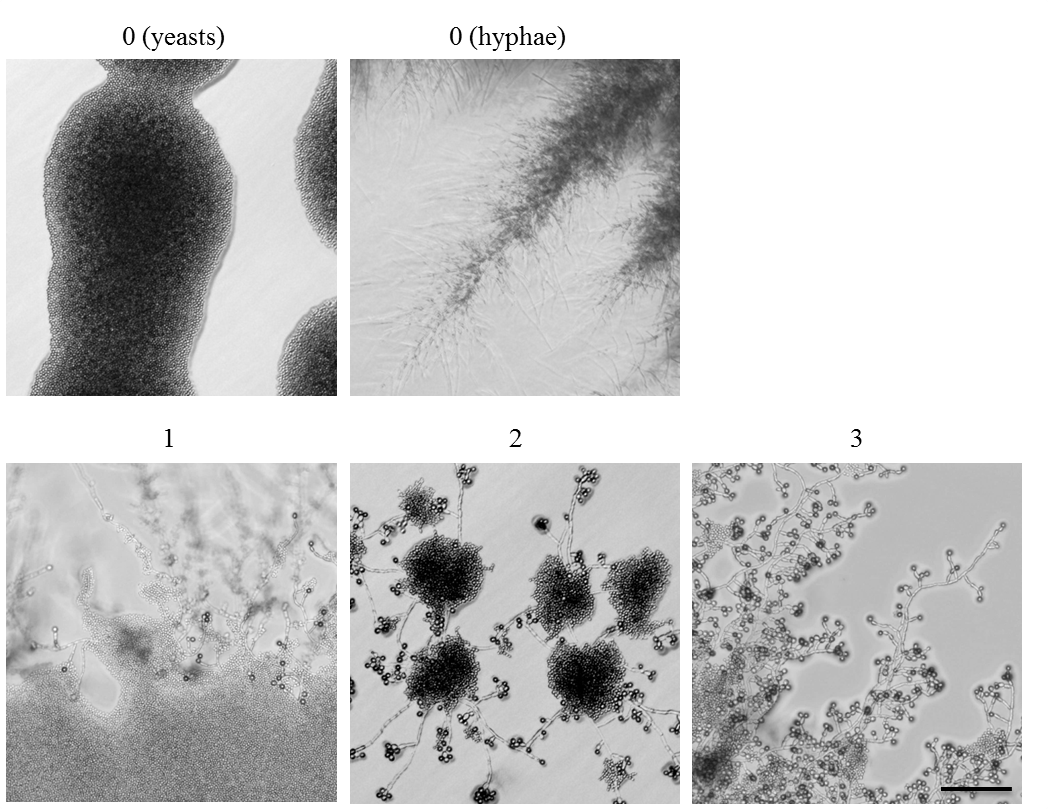
 Figure S1**: Comparative colony morphologies of *Candida* strains corresponding to the score system in table 2.

All *Candida* strains were incubated for seven days in darkness colony morphology was examined microscopically. Morphologies were grouped according the quantity of produced chlamydospores. Scores of chlamydospore indices (CI) range from 0: no chlamydospores to 3: masses of chlamydospores. Production of hyphae was not considered separately. Scale bar: 100 µm.


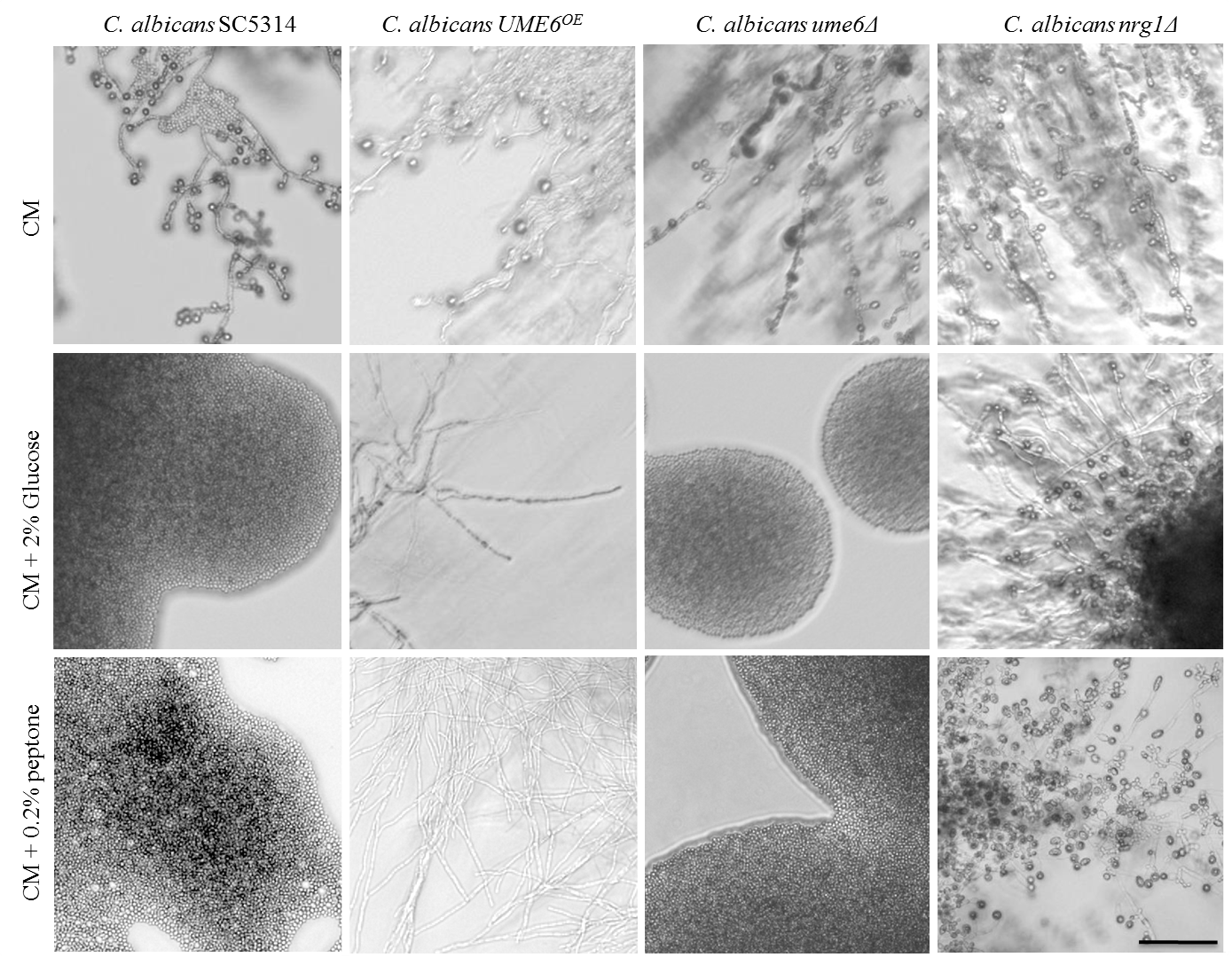


**Figure S2**: Role of *C. albicans* Ume6 during chlamydospore formation.

*C. albicans* strains were streaked onto pure and supplemented CM agar. Tor1-activity was specifically reduced by adding 20 nM rapamycin or 5 mM caffeine to the agar. On day 7 after incubation (27°C, darkness) colony morphology was examined microscopically. Neither overexpression of *UME6* could rescue chlamydospore formation nor did *UME6* gene deletion impair it. In contrast, *NRG1* deletion facilitated sporulation under all conditions tested. The scale bar represents 100 µm.


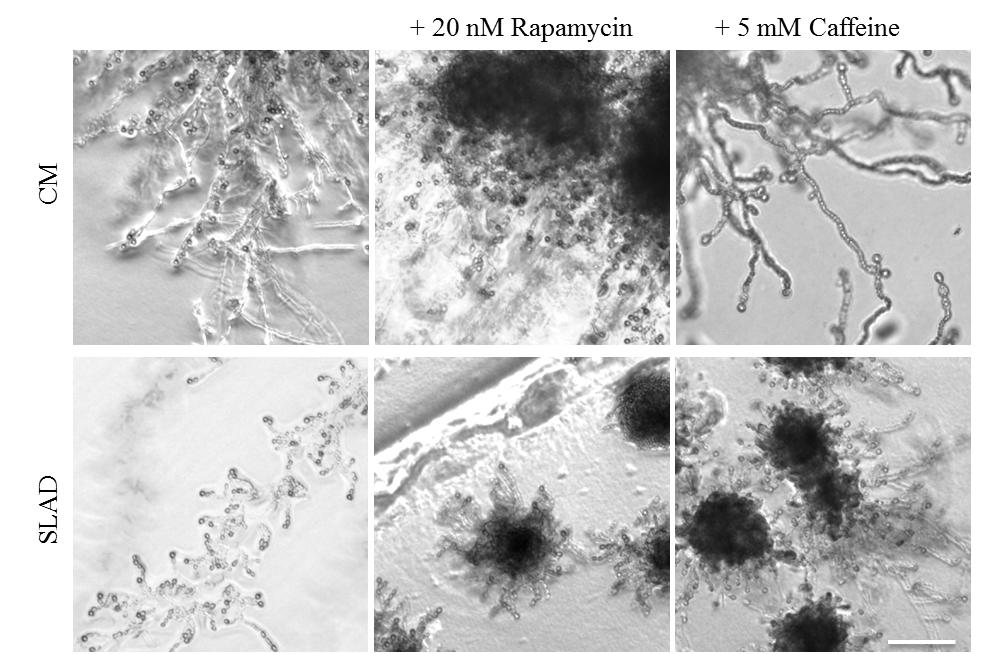


**Figure S3**: Chlamydospore formation by a *C. albicans* *nrg1Δ* deletion mutant

A *C. albicans* *nrg1Δ* strain was streaked onto CM and SLAD (YNB + 2% glucose) agar. Tor1-activity was specifically reduced by adding 20 nM rapamycin or 5 mM caffeine to the agar. On day 7 after incubation (27°C, darkness) colony morphology was examined microscopically. The gene *NRG1* gene deletion was epistatic to the inhibition of TOR signaling and led to proper sporulation. The scale bar represents 100 µm.


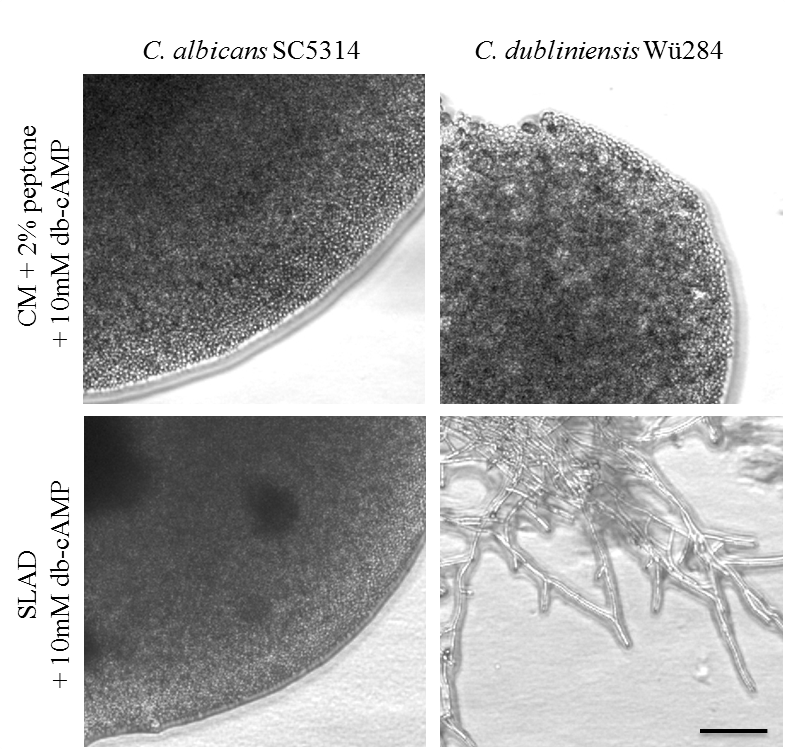


**Figure S4**: Addition of 10 mM db-cAMP cannot rescue chlamydospore formation under repressive conditions.

*C. albicans* and *C. dubliniensis* wild type strains were incubated for 7 days on 2% peptone-containing CM agar and SLAD agar in presence of 10 mM db-cAMP. Db-cAMP supplementation did not induced chlamydospore formation in any condition, but rather blocked sporulation by *C. dubliniensis* on SLAD agar. The scale bar represents 100 µm.


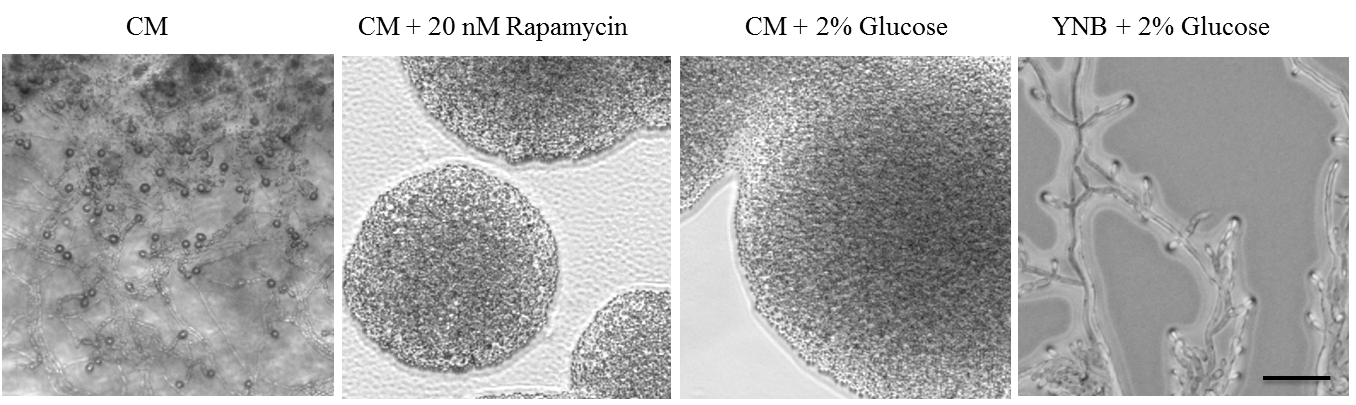


**Figure S5** Chlamydospore formation by a *C. albicans* *pde2Δ* deletion mutant

A *C. albicans* *pde2Δ* strain was streaked onto CM and SLAD (YNB + 2% glucose) agar. Chlamydospores were formed on CM agar but production was inhibited by adding 20 nM rapamycin or 2% glucose to CM. In comparison to *C. albicans* wild type strain, growth on SLAD agar induced filamentation of a *pde2Δ* mutant but no chlamydospores were observed. The scale bar represents 100 µm.
